# Adiponectin, ALT and family history as critical markers for the development of type 2 diabetes in obese Japanese children

**DOI:** 10.1002/edm2.178

**Published:** 2020-08-28

**Authors:** Yuki Yasuda, Nobuka Miyake, Hisafumi Matsuoka, Shigetaka Sugihara

**Affiliations:** ^1^ Department of Pediatrics Tokyo Women's Medical University Medical Center East Tokyo Japan

**Keywords:** adiponectin, metabolic syndrome, type 2 diabetes mellitus

## Abstract

**Aims/Introduction:**

An association between the pathogenesis of type 2 diabetes mellitus (T2D) and that of metabolic syndrome (MS) in obese children has been suggested. We clarified the critical markers for the development of T2D in obese Japanese children.

**Methods:**

One hundred and seven obese children who visited our outpatient clinic were enrolled in this study. The obese subjects were divided into 3 groups: Group A, T2D (n = 19); Group B, MS but not T2D (n = 19); and Group C: non‐T2D, non‐MS (n = 69). In all the subjects, a biochemical examination was performed and the serum adiponectin and leptin levels were measured. Visceral adipose tissue (VAT) and subcutaneous adipose tissue (SAT) were measured using computed tomography images.

**Results:**

Group A tended to have higher VAT values and VAT/SAT ratios and lower leptin and adiponectin levels, compared with Groups B and C. In Group A, the alanine aminotransferase (ALT) level was significantly higher and the aspartate aminotransferase (AST)/ALT ratio was significantly lower than in Group C. A receiver operating characteristic (ROC) analysis showed that the optimal cut‐off point for adiponectin was 6.4 μg/mL (AUC = 0.859). The cut‐off points for ALT, the AST/ALT ratio and VAT were 35 IU/L (AUC = 0.821), 0.85 (AUC = 0.794) and 78 cm^2^ (AUC = 0.713), respectively. Group A had a significantly higher frequency of a family history of T2D than Group B.

**Conclusions:**

Our study revealed that the adiponectin level, ALT level, AST/ALT ratio, VAT value and a family history of T2D may be critical characteristic markers for T2D among obese Japanese children.

## Graphical abstract

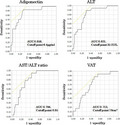

## 1 INTRODUCTION

The incidence of overweight and obese children has been increasing worldwide over the past several decades.^1^, ^2^, ^3^ Additionally, health problems caused by obesity, such as type 2 diabetes mellitus (T2D) and metabolic syndrome (MS), have become a serious problem.^1^, ^2^ In Japanese children, the incidence of obesity tripled between the 1970s and around 2000, although a tendency towards a slight decrease was subsequently reported. However, approximately 10% of children continue to be obese, with a per cent overweight (POW) of more than 20%, and the rate of severe obesity with a POW of more than 50% has not decreased.^4^

Metabolic syndrome in adults is defined as a clustering of cardiovascular and T2D risk factors.^1^, ^2^ An association between the pathogenesis of T2D and that of MS has been suggested in obese children.^5^, ^6^ Insulin resistance is also a major pathological factor in MS and T2D in obese children.^5^, ^6^ However, all obese children with MS do not always develop T2D. Our hypothesis is that the onset of T2D may require some additional factors in obese children that differ from those required for the onset of MS. We compared the characteristics of fat accumulation, blood chemistry and adipocytokine levels among obese T2D children and obese MS children without T2D as well as obese children with neither T2D nor MS. We then analysed the threshold value of the risk factors for T2D in obese Japanese children.

## 2 MATERIALS AND METHODS

### 2.1 Subjects and methods

The study subjects included 107 children and adolescents between the ages of 7 and 17 years who visited our outpatient clinic (Tokyo Women's Medical University Medical Center East, Tokyo, Japan) from 2001 to 2013.

First, we performed a detailed interview of the patients and their parents and obtained information such as a past history and the family history. If a subject had a first‐ or second‐degree relative with a diagnosis of T2D, the subject was regarded as having a positive family history.

The height, weight, waist circumference (WC) and blood pressure of each subject were measured. The per cent overweight (POW) is generally used to evaluate and diagnose childhood obesity in Japan.^4^ The POW is calculated using the following formula: POW (%) = (measured weight − standard weight)/standard weight × 100. The standard weight is the age‐ and sex‐specific weight for height based on data from the Annual Report of School Health Statistics 2000 from the Ministry of Education, Culture, Sports, Science and Technology, Japan.^4^ The criteria for obesity are POW ≧ 20% in school‐age children. A 20% POW may be almost equivalent to the 90th percentile of BMI in average height and weight children. A POW of ≧20% is defined as obesity, and ≧50% is defined as severe obesity. Measurement of the WC was performed in a standing position at the umbilical level.^4^

Blood specimens were collected in the morning after an overnight fast, and the aspartate aminotransferase (AST), alanine aminotransferase (ALT), triglycerides (TG), total cholesterol, high‐density lipoprotein cholesterol (HDL‐C), uric acid and fasting blood glucose levels (FBS) were measured using an automated analyser (Labospect 008; Hitachi High‐Technologies). HbA1c was measured by HPLC and expressed as the National Glycohemoglobin Standardization Program value. Immunoreactive insulin (IRI) was measured using chemiluminescent enzyme immunoassay (CLEIA). The leptin level was measured using a double‐antibody radioimmunoassay method. The adiponectin level was measured using the enzyme‐linked immunosorbent assay (ELISA) method (Otsuka Pharmaceutical Co., Ltd) between 2001 and March 2010 and the latex particle‐enhanced turbidimetric immunoassay method (Mitsubishi Chemical Co., Ltd)^7^ between April 2010 and 2013. The correlation between these two methods was significantly strong: *Y* = 0.973*X* + 0.075 (X: ELISA, Y: Latex, *r* = 0.983).

Visceral adipose tissue (VAT) and subcutaneous adipose tissue (SAT) levels were measured using cross‐sectional computed tomography images at the umbilical level.

T2D was diagnosed according to the criteria of the Japan Diabetes Society (JDS) and the American Diabetes Association.^8^, ^9^ MS was diagnosed according to the MS diagnostic criteria for Japanese children established by the Ministry of Health, Labor and Welfare study group.^4^ MS in children aged 6‐15 years was defined as WC ≥ 80 cm or waist‐to‐height ratio ≥ 0.5; TG ≥ 120 mg/dL and/or HDL ≤ 40 mg/dL; systolic BP ≥ 125 mm Hg and/or diastolic BP ≥ 70 mm Hg; and/or FBS ≥ 100 mg/dL. MS was defined as two or three positive risk factors (dyslipidemia, hyperglycaemia and hypertension) in addition to a high WC in obese children.

The subjects were divided into 3 groups: Group A, obese children with T2D (n = 19); Group B, obese children with MS but not with T2D (n = 19); and Group C, obese children with neither T2D nor MS (n = 69). The results of each examination were then compared among these three groups. Furthermore, a receiver operating characteristic (ROC) analysis was performed for each data as an analysis of the risk factors from obesity to T2D.

### 2.2 Statistical analysis

The results were expressed as the mean ± SD or median (range). The Fisher exact test and *χ* ^2^ tests were applied to a two‐by‐two contingency table. Clinical data among the three groups were compared using the Kruskal‐Wallis test. A ROC analysis was performed to determine the threshold value of the risk factors for the onset of T2D in obese children. *P* values of <.05 were considered as denoting statistical significance. All the statistical analyses were performed using JMP pro 12 (SAS Institute).

## 3 RESULTS

The clinical characteristics of the three groups of subjects are shown in Table 1. No significant differences in male/female ratio, age, POW (%) or SAT area (cm^2^) were observed among the three groups. Height, body weight, VAT area (cm^2^) and the VAT/SAT ratio were significantly higher in Group A (T2D) than in Group C (non‐T2D, non‐MS), but no significant difference was seen between Group A and Group B (MS) (Table 1).

**TABLE 1:** Clinical characteristics of the three groups of subjects

	A: T2D	B: MS	C: non‐T2D, non‐MS	*P*	*P*
	(n = 19)	(n = 19)	(n = 69)	A vs B	A vs C
Male (%)	53%	78.9%	43%	NS	
Age (y)	12.7 (7‐15)	12.1 (8‐15)	10.2 (7‐17)	NS	NS
Height (cm)	159.1 ± 11.0	159.2 ± 12.9	150.0 ± 12.9	NS	<.05
BW (kg)	74.4 ± 16.5	77.8 ± 19.8	57.5 ± 18.3	NS	<.05
Per cent Overweight (%)	49.8 ± 18.7	57.3 ± 18.4	47.9 ± 19.3	NS	NS
SAT (cm^2^)	269.3 ± 78.2	306.8 ± 95.4	242.8 ± 88.2	NS	NS
VAT (cm^2^)	104.1 ± 46.5	92.2 ± 28.4	70.7 ± 28.1	NS	<.01
VAT/SAT	0.397 ± 0.169	0.316 ± 0.111	0.304 ± 0.105	NS	<.05

Abbreviation: NS: not significant.

Table 2 compares the components of MS among the three groups. No significant differences in waist circumference, waist/height ratio, serum TG levels, diastolic BP or the number of positive MS diagnostic factors were seen between Groups A and B. Of note, Group A had significantly lower HDL‐C levels (*P* < .0001), higher FBS levels (*P* < .0001) and lower systolic BP values (*P* < .0001) than Group B (Table 2).

**TABLE 2:** Comparison of metabolic syndrome factors among three groups

	A: T2D	B: MS	C: non‐DM, non‐MS	*P*	*P*
	(n = 19)	(n = 19)	(n = 69)	A vs B	A vs C
Waist Circumference (cm)	94.3 ± 10.6	98.0 ± 10.2	85.0 ± 18.3	NS	<.05
Waist/Height	0.59 ± 0.06	0.62 ± 0.05	0.39 ± 0.09	NS	<.0001
TG (mg/dL)	178.7 ± 116.6	137.1 ± 101.3	82.5 ± 30.7	NS	<.0001
HDL‐C (mg/dL)	46.2 ± 7.2	52.8 ± 13.2	56.5 ± 12.4	<.0001	NS
FBS (mg/dL)	158.9 ± 56.2	101.0 ± 7.9	92.4 ± 7.5	<.0001	<.0001
SBP	114.2 ± 11.9	131.9 ± 14.9	113.1 ± 11.8	<.0001	NS
DBP	68.1 ± 11.1	75.1 ± 8.7	64.4 ± 8.8	NS	NS
Number of positive MS factors	2.8 ± 1.0	3.2 ± 0.4	1.1 ± 0.7	NS	<.0001

**FIGURE 1:**
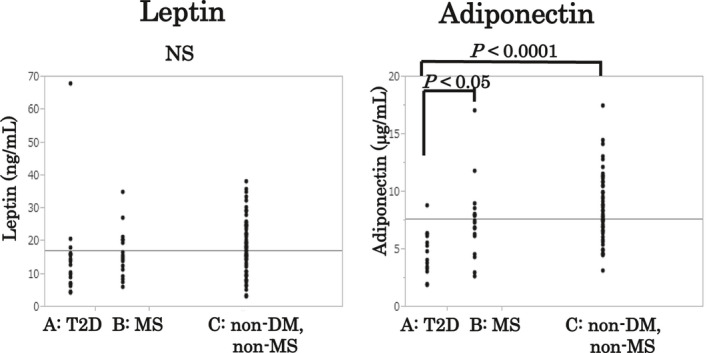
Comparison of leptin and adiponectin among three groups of obese children. Group A tended to have lower leptin levels and had significantly lower adiponectin levels than Group B (*P* < .05) and Group C (*P* < .0001)

Group A tended to have higher VAT values and VAT/SAT ratios (Table 1), and lower leptin levels than Groups B or C (Table 3, Figure 1). Group A had significantly lower adiponectin levels, compared with Group B (*P* < .05) and Group C (*P* < .0001; Table 3, Figure 1).

**TABLE 3:** Comparison of ALT, leptin, adiponectin, family history and other indexes among three groups

	A: T2D	B: MS	C: non‐DM, non‐MS	*P*	*P*
	(n = 19)	(n = 19)	(n = 69)	A vs B	A vs C
HbA1c (%)	8.31 ± 1.90	5.17 ± 0.29	4.96 ± 0.29	<.0001	<.0001
IRI (μU/mL）	24.3 ± 2.73	22.0 ± 2.73	17.3 ± 1.43	NS	NS
UA (mg/dL)	6.9 ± 2.0	6.8 ± 1.9	5.48 ± 1.4	NS	.0003
ALT (IU/L)	89.1 ± 63.9	56.3 ± 52.1	34.8 ± 33.5	NS	<.05
ALT ≧ 35 (IU/L)	16 (84.2%)	9 (47.4%)	17 (24.6%)	<.05	<.0001
ALT/AST > 1	19 (100%)	14 (73.4%)	32 (46.4%)	NS	<.0001
Leptin (ng/mL)	15.2 ± 14.0	16.02 ± 6.97	17.88 ± 8.13	NS	NS
Adiponectin (µg/mL)	4.65 ± 1.92	7.35 ± 3.27	8.32 ± 2.71	<.05	<.0001
Family history (%)	15 (79.0%)	9 (47.4%)	22 (31.9%)	<.05	<.05

In Group A, the ALT levels were significantly higher (*P* < .05) and the AST/ALT ratios were significantly lower (*P* < .0001) than those in Group C (Table 3).

**FIGURE 2:**
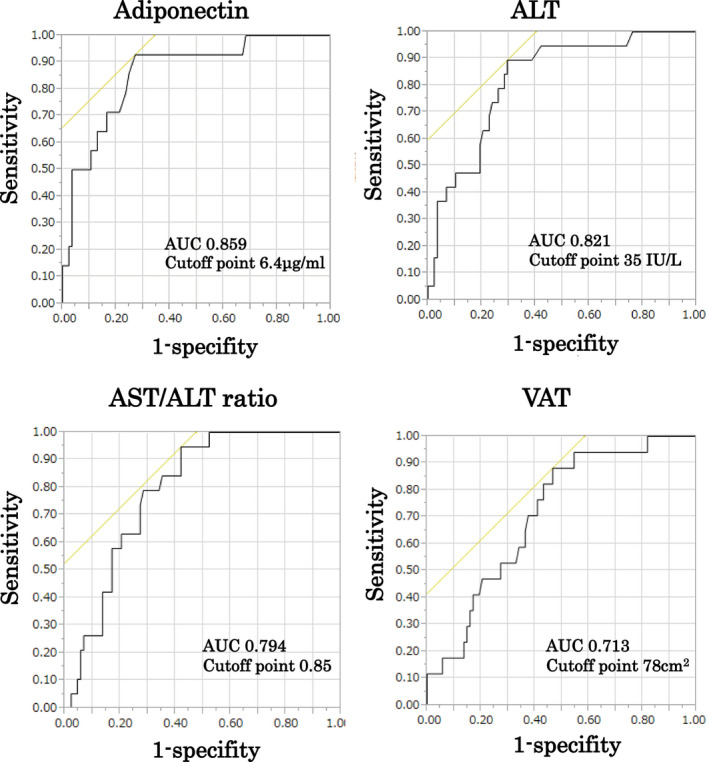
ROC analysis for the onset of T2D. The ROC analysis for the onset of T2D showed that the optimal cut‐off point for adiponectin was 6.4 μg/mL (AUC = 0.859), while the optimal cut‐off points for ALT, the AST/ALT ratio and VAT were 35 IU/L (AUC = 0.821), 0.85 (AUC = 0.794) and 78 cm^2^ (AUC = 0.713), respectively

The ROC analysis for the onset of T2D showed that the optimal cut‐off point for adiponectin was 6.4 µ g/mL (AUC = 0.859), while the optimal cut‐off points for ALT, the AST/ALT ratio and VAT were 35 IU/L (AUC = 0.821), 0.85 (AUC = 0.794) and 78 cm^2^ (AUC = 0.713) (Figure 2).

In Group A, 15 (79%) of the 19 subjects had a positive family history of T2D. On the other hand, 9 (47%) of the 19 subjects had a positive family history of T2D in Group B. Group A had a significantly higher frequency of a positive T2D family history than Group B (*P* < .05) and C (*P* < .05; Table 3).

## 4 DISCUSSION

Our study demonstrated that obese Japanese children with T2D (Group A) had a significantly lower adiponectin level but did not have a higher leptin level (Figure 1).

In children, as in adults, the accumulation of VAT is important in the pathology of MS. The accumulation of VAT increases insulin resistance and increases the risk of impaired glucose tolerance, abnormal lipid metabolism, increased BP and the development of NAFLD (nonalcoholic liver disease).^6^, ^10^, ^11^, ^12^ And worsening glucose tolerance leads to the development of T2D.

Various adipocytokines secreted from the adipose tissues are involved in the pathogenesis of T2D and MS.^10^ Leptin and adiponectin are associated with insulin resistance and MS in children and show anti‐inflammatory effects.^10^ Adiponectin is regarded as a ‘good’ adipocytokine with antidiabetes effects and anti‐arteriosclerotic effects. Additionally, adiponectin is strongly related to insulin sensitivity, so a decrease in adiponectin causes an increase in insulin resistance. Previous reports have shown that serum adiponectin levels are inversely correlated with insulin resistance and parameters of MS in both adults and children.^13^, ^14^, ^15^, ^16^ Hypoadiponectinemia is correlated with the development of T2DM in adults.^10^ We previously demonstrated that the plasma PAI‐1 levels, but not the leptin levels, of obese Japanese children were also significantly correlated with insulin resistance indexes, such as the immunoreactive insulin (IRI), HOMA‐R and QUICKI values.^17^

In a study examining Pima Indian children, the plasma adiponectin concentrations were negatively correlated with the percentage of body fat and the fasting plasma insulin concentrations.^15^ Asayama et al also reported that adiponectin levels were significantly lower in obese Japanese children and were inversely correlated with the VAT area.^16^

Visceral fat accumulation may induce changes in adipocytokines, such as adiponectin, PAI‐1 and oxidative stress. In other words, the overproduction of inflammatory cytokines and PAI‐1 results in inflammatory and thrombogenic properties and a deficiency of adiponectin. Fibrosis is also caused by an increase in oxidative stress.^18^

When VAT accumulation becomes excessive, ‘ectopic fat accumulation’ is thought to begin in a wide variety of sites including the liver, skeletal muscle and pancreas. A higher ALT and a lower AST/ALT ratio indicate the presence of NAFLD, which means ectopic fat accumulation in obese children. Our results indicate that Group A may have NAFLD, as ectopic fat accumulation was observed more frequently than in Groups B and C.

Adiponectin acts on the liver and activates the transcription factor PPARα, thereby enhancing fatty acid β oxidation in hepatocytes and reducing neutral fat in hepatocytes. Therefore, the decrease in adiponectin suggests the possibility of exacerbating the pathology of NAFLD.^19^, ^20^ A large‐scale survey of Japanese adult medical examination data showed a significant correlation between visceral obesity and liver enzyme (ALT) and a negative correlation between adiponectin levels and liver function.^21^

Furthermore, we performed a ROC analysis for the onset of T2D. Adiponectin had the highest AUC, and the optimal cut‐off point was 6.4 µg/mL. Significant results were also obtained for the ALT level, AST/ALT ratio and VAT value. These findings also suggest that a reduction in adiponectin is deeply involved in the development of T2D in obese Japanese children.

Our results indicate that obese Japanese children with T2D tend to have larger amounts of VAT and ectopic fat, such as NAFLD, and lower levels of adiponectin than obese children with MS but without T2D. Of note, the leptin/adiponectin ratio was not a useful marker of T2D in obese Japanese children in this study. There have been many reports that the leptin/adiponectin ratio is more useful than either leptin or adiponectin alone as an index for evaluating MS.^22^, ^23^ In our current study, the leptin/adiponectin ratio was not a significant indicator because both the leptin level and the adiponectin level were relatively low in Group A, compared with Groups B and C (Figure 1).

A family history of T2D was shown to be another important factor for the onset of T2D in obese Japanese children. Oh et al^24^ showed that obese Korean children and adolescents with a family history of T2D were more insulin resistant and had lower serum adiponectin levels, compared with an obese group without a family history of T2D. Another study has also shown that a family history of T2D in white youths was associated with reduced insulin sensitivity.^25^

## 5 CONCLUSION

The progression of T2D from MS may be strongly linked to an increase in VAT, a decrease in adiponectin and an increase in ectopic fat accumulation, such as NAFLD, in obese Japanese children. The reduction in the adiponectin level, higher level of ALT and a family history of T2D may be the most important factors for the development of T2D. These risk factors may be very useful for the early intervention and prevention of T2D in obese Japanese children.

## AUTHOR CONTRIBUTIONS

Y.Y and S.S contributed to the conception and design of the study. YY and NM contributed to the data analysis. All authors contributed to the acquisition and/or interpretation of the data. Y.Y wrote the manuscript. All authors critically read the manuscript, suggested revisions and approved the final version of the manuscript.

## Ethical approval

This study was approved by the review board of Tokyo Women's Medical University and was conducted in accordance with the ethical guidelines and regulations laid out in the Declaration of Helsinki (approval number: 1413). Written informed consent was obtained from each of the subjects or their parents.

## Data Availability

The data sets used and/or analysed during the current study are available from the corresponding author on reasonable request.
